# The Dollar and Blood Aristocracy

**Published:** 1856-07

**Authors:** 


### THE DOLLAR AND BLOOD ARISTOCRACY.

Our first visit to London found us in private lodgings—No Three, Spring Gardens. Early next morning, we sauntered into St. James' Park, close by, and on inquiring the ownership of a very common, unpainted, dingy looking dwelling, some three stories high, if we remember well, we learned it was the residence of "QUEEN VICTORIA." Not far from it was an old cow, tied to a tree, around which were congregated a number of nurses, each with a baby and a mug, going up in turn to get their share of pure and undiluted milk. We cannot tell how wide our unsophisticated mouth opened just at that moment, but it was considerable, if not more so. Our ideas of a Palace, formed away out yonder in the grazing pastures of Kentucky, a long, long time ago, were, that it could not be much less than a dozen stories high, with all sorts of towers and gilded things to match; and as for such a vulgar article as a cow being within miles of it, we never dreamed of such a thing, but the reality was as we have stated. We cannot imagine that Queen Victoria feels at all lowered in occupying for herself, and rearing her children in a common three story brick house. It is on her blood and birth that she relies. Her character and her position are her pride. Yes! the heirs of an untoiled for income of hundreds of thousands a year are content to occupy a three story brick house. It is the recently rich, the newly elevated, who revel in glare, and glitter and show. It is the brewer's wife, whose whole ambition is to get into society. It is the butcher's daughter, who dresses violently. Those whose positions have been undoubted for generations, man, woman or child, would not be considered "anybody in particular" in a walk along Broadway, from anything that pertained to dress, but an observer detects it in a moment; there is an "air," there is a "presence" about them, which needs no interpreter. On the other hand, what violent transitions are there between the "*superbly dressed woman*" and her plebeian face; between the splendid "*turn-out*" and its pug-nosed occupant; between the band-box exquisite, or the "*flushed*" black-leg, and the impudent stare, or cowering look, which are the inseparable attendants of the consciously degraded, the world over.

Well! passing up our own Fifth Avenue, or down Fourteenth street, or around Union Square, or Madison Park, or Murray Hill, we find multitudes of palatial residences, as far superior in their external appearance to the Palace of St. James as one can well imagine. A residence costing Fifty Thousand Dollars is a common thing in the above-named localities. The oak carvings, beautiful and chaste they are, of a single parlor in University Place, cost three thousand dollars; and there are several dwellings, the adornments of single rooms of which have cost fifteen, twenty, and even as high as thirty thousand. More than one residence in New-York has cost, with its furnishings, not much less, exaggerations lain aside, than two hundred thousand dollars! These men have made their own money by severe industry and patient assiduity in business; and we are rather fearful that we are not a little impertinent in making any special remark about the outlay of what is their own. The fact is, we like a generous expenditure of one's means; it elevates the man, and has an elevating influence on all about him, his servants, his tradesmen, his friends, his children, and all. It is your poor, pitiful, narrow-hearted, close-fisted, mean-minded miser, who never parts with a dollar but with a pain; that is the kind of man on whom we look with unpitying contemptuousness. But for all this, we have often inquired whether any parent, wisely kind, can bring up his children in a style and manner of living which he cannot leave them the means of sustaining. There are men so stupid, that their heads cannot be turned by any elevation; no unanticipated heights make them dizzy. But to descend safely, to do it in youth, to begin married life with a declivity, who is equal to it? not one in many thousands. And what is the result? ye merchant princes, ye successful stock-jobbers, ye retired bankers of New-York, Philadelphia and Boston, we repeat the inquiry. What is the necessary result, as a general rule, as affecting the destinies of your children, who cannot, if they go out into the world, sustain the style of their father's house? The boys decline marriage, and with it give up, at one fell swoop, the purities, the joys, the elevations of domestic life. The next thing is to join some "Club," where introductions are soon made to the cigar, the wine-cup, the chess-board, the coarse jest, the loud laugh, the bacchanal song, the rail against "*Puritanism*," the Sabbath drive, or yachting, or sauntering. Then comes apace things said and done, which the pure ears of beauty can never hear, nor eyes see, nor hearts conceive, without mantling the young cheek with shame.

As for your daughters, so loving and so loved to you, what is their future? To marry "upwards," as the world calls it, they cannot. Nor can they marry men, except in rare instances, who can even *maintain* the style of living in their father's home. They must therefore marry downwards, or not marry at all, and not marrying, may almost as well be dead. In a few years, and father and mother will be gone. Brothers have formed other ties. One by one of the associates of other years is lost from their visiting list, by removal, or marriage, or death. Every year leaves them more and more lonely, more and more neglected; and soon thereafter the great world loses sight of them; their very names are only now and then mentioned, while all this time they are consuming themselves with sad memories, and anon pass unwept into a forgotten grave.

Therefore, we say to *wealthy parents*, *if you truly love your children, live in that style which you can enable each one of them to sustain.*

